# Increased Risk of End-Stage Kidney Disease After Traumatic Amputation: Nationwide Cohort Study

**DOI:** 10.3390/healthcare13010080

**Published:** 2025-01-04

**Authors:** Jung Eun Yoo, Bongseong Kim, Won Hyuk Chang, Kyungho Lee, Hye Ryoun Jang, Kyungdo Han, Dong Wook Shin

**Affiliations:** 1 Department of Family Medicine, Healthcare System Gangnam Center, Seoul National University Hospital, Seoul 06236, Republic of Korea; ujungeun@gmail.com; 2 Department of Family Medicine, Seoul National University College of Medicine, Seoul 03087, Republic of Korea; 3 Department of Internal Medicine, The Catholic University of Korea, Seoul 06591, Republic of Korea; qhdtjd12@gmail.com; 4 Department of Physical and Rehabilitation Medicine, Center for Prevention and Rehabilitation, Heart Vascular Stroke Institute, Samsung Medical Center, Sungkyunkwan University School of Medicine, Seoul 06351, Republic of Korea; wh.chang@samsung.com; 5 Division of Nephrology, Department of Medicine, Samsung Medical Center, Sungkyunkwan University School of Medicine, Seoul 06351, Republic of Korea; kidney.lee@samsung.com (K.L.); hyeryoun.jang@samsung.com (H.R.J.); 6 Department of Statistics and Actuarial Science, Soongsil University, 369 Sangdo-ro, Dongjak-gu, Seoul 06978, Republic of Korea; 7 Supportive Care Center/Department of Family Medicine, Samsung Medical Center, Sungkyunkwan University School of Medicine, Seoul 06351, Republic of Korea; 8 Department of Clinical Research Design & Evaluation, Samsung Advanced Institute for Health Science & Technology (SAIHST), Sungkyunkwan University, 81 Irwon-Ro, Gangnam-gu, Seoul 06351, Republic of Korea

**Keywords:** trauma, amputation, disability, renal function, end-stage kidney disease

## Abstract

Background: Amputation confers disabilities upon patients and is associated with substantial cardiovascular and metabolic morbidity and mortality. We aimed to compare the incidence of end-stage kidney disease (ESKD) between individuals with amputation and the general population. Methods: A population-based retrospective cohort study was performed using the Nationwide Health Insurance Service database for the period between 2010 and 2018. A total of 24,925 individuals with amputation were included with a ratio of 1:3 age- and sex-matched controls. A Cox proportional hazards regression analysis was used to calculate the risk of ESKD among amputees. Results: During a mean follow-up period of 4.3 years, there were 40 incident ESKD cases (0.4 per 1000 person-years) among individuals with amputation. Individuals with amputation showed a higher risk of ESKD (adjusted HR [aHR] of 1.75, 95% confidence interval [CI] of 1.20–2.54) compared with matched controls. The risk was further increased in those with mild disability (aHR of 1.41, 95% CI of 0.51–3.87) and severe disability (aHR of 8.22, 95% CI of 2.99–22.61). When considering the levels of amputation, the association was apparently more prominent in proximal than distal amputation, in particular for proximal upper limb amputation (aHR of 17.90, 95% CI of 4.37–73.40). Conclusions: Individuals with amputation were at a significantly greater risk of ESKD than the general population, particularly subjects with severe disability and proximal amputation. Our data suggest that amputations should be added to the list of risk factors for the development of chronic kidney disease.

## 1. Introduction

Amputation causes disability and severely impairs patient mobility and physical function. The leading cause of amputation varies from region to region. Peripheral vascular disease and diabetes are commonly identified as the leading causes of amputation in high-income countries [1]. However, in many low- and middle-income countries, trauma remains the primary cause of amputation [1]. Individuals with traumatic amputations typically sustain their injuries at relatively young age and have a long life expectancy [2], emphasizing the need for longitudinal health care.

Chronic kidney disease (CKD) has become a major global public health problem, and as a major non-communicable disease, is associated with adverse clinical outcomes. Patients with CKD stages 3–5 account for nearly half of CKD cases worldwide, and most gradually develop end-stage kidney disease (ESKD) and require renal replacement therapy due to the progressive nature of CKD stages 3–5 [3]. The majority of CKD patients have diabetes, hypertension, and cardiovascular disease, driven by the reciprocal relationship among these conditions which complicates relevant treatment [4].

Recently, individuals with amputation were shown to have significantly worse cardiovascular and metabolic issues [5]. Even when an amputation occurs as the result of an accident or traumatic injury, individuals with amputation reportedly are 2-fold more at risk of developing metabolic disorders including obesity, hypertension, glucose intolerance, and hyperlipidemia compared with the general population [6], which can be a risk factor for CKD. In addition, decreased survival among amputees with ESKD has been reported in several studies [7,8,9,10,11]. However, most of the focus has been on dysvascular amputation [9,10,11] or the prognosis of amputees who already have ESKD [7,8,9,10,11]. In cases of dysvascular amputation, patients are already at a high risk of ESKD. Thus, information on the effects of amputation on long-term renal function is lacking. Therefore, we conducted a nationwide retrospective study to investigate the risk of incident ESKD following amputation compared with the general population. In addition, we assessed how the severity of disability resulting from the amputation and the levels of amputation affect the association between amputation and ESKD.

## 2. Materials and Methods

### 2.1. Data Source and Study Setting

The present study was based on the database provided by the National Health Insurance Service (NHIS) in Korea, which is a single universal insurer in Korea. The NHIS database contains demographic data and links the data to health care claims based on medical claims according to the International Classification of Diseases, 10th edition (ICD-10), codes. Because the NHIS provides at least biennial health screenings for all insured Koreans, the NHIS database also contains health screening data, including self-administered health questionnaires, anthropometric measurements, and laboratory tests [12].

### 2.2. Study Population

Initially, 59,392 individuals with an amputation that occurred between 2010 and 2018 were identified. The individuals with amputation were selected based on ICD-10 codes as follows: Z89 (acquired absence of limb), S48 (traumatic amputation of shoulder and upper arm), S58 (traumatic amputation of elbow and forearm), S68 (traumatic amputation of wrist, hand, and finger), S78 (traumatic amputation of hip and thigh), S88 (traumatic amputation of lower leg), and S98 (traumatic amputation of ankle and foot) [13,14]. Among the patients, 27,832 individuals with amputation who had had a health screening within 2 years before amputation were included. Participants were excluded for the following reasons: (1) being <20 years of age (n = 22); (2) a diagnosis of diabetes with complications (n = 456); (3) an amputation due to vascular causes such as a foot ulcer (n = 44), peripheral arterial disease (e.g., Buerger’s disease, n = 140), or occlusive peripheral arterial disease (n = 1002); (4) a diagnosis of ESKD before amputation (n = 25) or within 1 year after amputation (1-year lag, n = 140); and (5) any missing information (n = 1078). Finally, a total of 24,925 individuals with amputation were included in this study.

**Figure 1:**
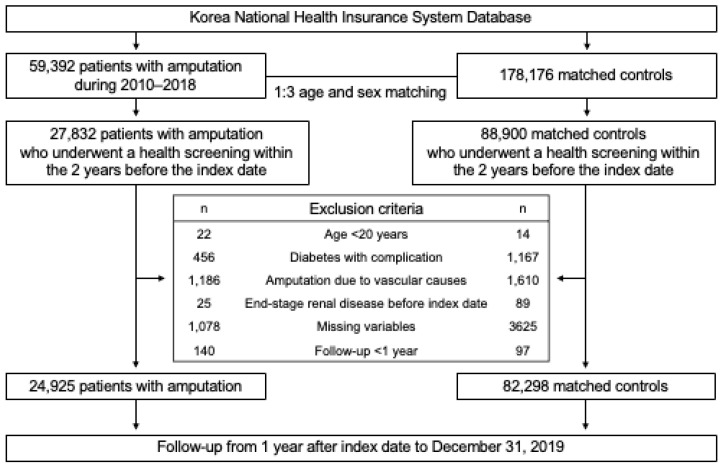
Flow chart of the study population.

For the selection of the control group, a 1:3 matching process based on age and sex was applied. The matched controls were assigned an index date that aligned with the amputation date of their corresponding individuals. After applying the same exclusion criteria, 82,298 matched controls were included in the analysis. The study population selection process is illustrated in Figure 1.

### 2.3. Amputation: Disability and Levels of Injury

To consider the effect of disability, the disability grade registered in the National Disability Registry (NDR) of the Ministry of Health and Welfare was used. In Korea, people with disability are registered to obtain social benefits from the government. To diagnose a disability of the extremities due to amputation, physical examinations and X-ray tests are required [15]. Disability grades are determined based on the disability severity, ranging from 1 being the most severe to 6 being the mildest. In general, the amputation of proximal sites is a severe level of disability (Tables S1 and S2). For example, patients with grade 1 amputation have both arms amputated above the wrist joint (upper extremities) or both legs above the knee joint (lower extremities), and subjects with grade 6 amputation have one phalange amputated above the interphalangeal joint (upper extremities) or one phalange below the tarsometatarsal joint (lower extremities) [15]. In this study, patients who were registered in the NDR within 1 year after the index date were classified into the disability group and further divided into mild (grades 4–6) and severe (grades 1–3) disability groups. In addition, the levels of amputation were grouped as upper limb (above the elbow, between the elbow and wrist, and below the wrist) and lower limb (above the knee, between the knee and ankle, below the ankle).

### 2.4. Study Outcomes and Follow-Up

The study outcomes were newly diagnosed ESKD based on the ICD-10 codes during follow-up. ESKD was defined using a combination of ICD-10 codes (N18–19, Z49, Z94.0, and Z99.2), along with the initiation of renal replacement therapy and/or the receipt of kidney transplantation during hospitalization [16,17,18]. The Korean Health Insurance Review and Assessment Service covers all medical expenses for dialysis. Additionally, patients with ESKD are registered as special medical aid beneficiaries. As a result, all ESKD patients in the entire Korean population were eligible for inclusion, and data from all patients who started dialysis were analyzed. The treatment codes for dialysis or medical expense claims were O7011–O7020 or V001 for hemodialysis, O7071–O7075 or V003 for peritoneal dialysis, and R3280 for kidney transplantation. Individuals with no prior diagnosis of CKD who had a transplant or dialysis code on the same day as an acute renal failure code were excluded. Patients on continuous renal replacement therapy or acute peritoneal dialysis were also excluded. The cohort was followed for 1 year after the index date (lag period) until the date of ESKD diagnosis or the last follow-up date (31 December 2019), whichever occurred first.

### 2.5. Covariates

Sociodemographic information, including the age, sex, income level, and residential area, was considered as a potential confounder. Alcohol consumption was divided into three groups: none, mild to moderate (less than 30 g of alcohol/day), and heavy (30 g/day or more). The smoking status was recorded as never, a former smoker, or a current smoker. Regular physical activity was classified as either moderate-intensity exercise lasting over 30 min per session at least 5 days weekly, or vigorous-intensity exercise lasting more than 20 min per session at least 3 days weekly. The body mass index (BMI) was calculated by dividing the weight in kilograms by the square of the height in meters (kg/m^2^). Systolic and diastolic blood pressure readings were taken with participants seated following a minimum rest period of five minutes. Blood samples were obtained after participants fasted overnight. The estimated glomerular filtration rate (eGFR, expressed as mL/min/1.73 m^2^) was derived using the Modification of Diet in Renal Disease formula. Comorbid conditions were identified through claims data available before the screening date and health screening findings; (1) hypertension was defined based on the presence of claims with ICD codes I10–I13 or I15, the use of antihypertensive medications, or measured blood pressure levels at or exceeding 140/90 mmHg, (2) type 2 diabetes was determined by claims with ICD codes E11–E14, antidiabetic prescriptions, or a fasting glucose level equal to or above 126 mg/dL, and (3) dyslipidemia was defined using ICD code E78, the prescription of lipid-lowering agents, or total cholesterol levels equal to or greater than 240 mg/dL.

### 2.6. Statistical Analysis

To examine differences in the baseline characteristics, a descriptive analysis was performed, comparing individuals with amputation to their matched controls. A Cox proportional hazards regression analysis was utilized to determine hazard ratios (HRs) and 95% confidence intervals (CIs) for ESKD among individuals with amputation. Model 1 was unadjusted. Model 2 accounted for the age, sex, income level, place of residence, alcohol consumption, smoking status, regular physical activity, BMI, eGFR, and comorbidities (hypertension, type 2 diabetes, and dyslipidemia). Additionally, we compared the baseline characteristics of amputees who developed ESKD and those who did not. The risk factors of developing ESKD were examined using a multivariate Cox regression model. Statistical analyses were conducted using SAS software (version 9.4, SAS Institute Inc., Cary, NC, USA), with results considered significant at a *p* value < 0.05.

## 3. Results

### 3.1. Baseline Characteristics

The baseline characteristics of the study population are summarized in Table 1. Among individuals with amputation, the mean age was 54.2 years (standard deviation [SD] of 12.1 years), and 76.3% were male. Compared to the matched controls, individuals with amputation had a higher proportion of individuals in the lowest income category. A larger percentage of individuals with amputation resided in rural areas compared to matched controls. In terms of health behaviors, individuals with amputation had higher rates of heavy alcohol consumption and current smoking, while fewer engaged in regular physical activity. The baseline BMI and eGFR tended to be slightly higher in patients with amputation than their matched controls. Regarding comorbidities, individuals with amputation had higher prevalence rates of hypertension and type 2 diabetes compared to matched controls, while dyslipidemia was slightly less prevalent.

### 3.2. Risk of ESKD Among Patients with Amputation

**Figure 2:**
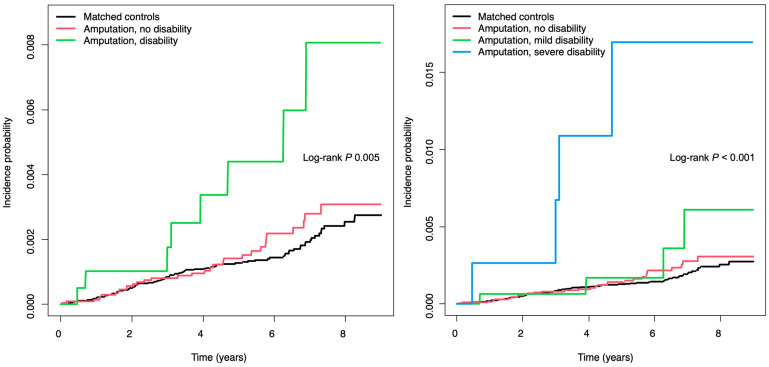
Kaplan–Meier survival analysis for the incidence of end-stage kidney disease in individuals with amputation compared with the matched controls.

During a mean follow-up period of 4.3 years (SD of 2.5 years), 40 incident ESKD cases (0.4 per 1000 person-years) were observed among individuals with amputation (Table 2). Compared with matched controls, individuals with amputation had a higher risk of ESKD (adjusted HR [aHR] of 1.75, 95% CI of 1.20–2.54) and the risk was further increased with the presence of disability (aHR of 2.42, 95% CI of 1.16–5.04). The Kaplan–Meier curves in Figure 2 show that the incidence probability of ESKD in patients with disability was higher than in matched controls (log-rank *p* = 0.005). Subjects with severe disability had the highest risk of ESKD (aHR of 8.22, 95% CI of 3.22–23.74). Based on the level of amputation, the risk of ESKD was higher in patients with amputation between the elbow and wrist (aHR of 17.90, 95% CI of 4.37–73.40) than in subjects with amputation below the wrist (aHR of 1.52, 95% CI of 1.01–2.27). In subjects with below-wrist amputation, the risk of ESKD increased with the severity of disability and was highest in those with severe disability (aHR of 6.91, 95% CI of 1.69–28.25) compared to matched controls.

Amputees who developed ESKD during the follow-up period were observed to have a significantly lower BMI, higher systolic blood pressure, elevated fasting glucose levels, and lower eGFR at baseline compared to those who did not develop ESKD (Table 3). Additionally, hypertension, type 2 diabetes, and dyslipidemia were more prevalent in this group. Hypertension (HR of 5.55, 95% CI of 2.00–15.39) and dyslipidemia (HR of 2.49, 95% CI of 1.19–5.21) were identified as independent risk factors for ESKD development.

## 4. Discussion

To the best of our knowledge, this is the first study in which the relative risk of ESKD among individuals with amputation was investigated and compared with the general population. Individuals with amputation had a higher risk of ESKD compared to matched controls, with the risk further increasing in those with disability and severe disability. In addition, the risk of ESKD was highest in patients with proximal amputation. Hypertension and dyslipidemia were independent risk factors for ESKD development in individuals with amputation.

Amputation leads to the crushing of muscles and other soft tissues, resulting in the release of potentially toxic intracellular components such as myoglobin into the systemic circulation. Consequently, amputation can cause acute kidney injury due to the direct effects of heme products and the tubular obstruction caused by myoglobin and urate crystals [19]. The uptake of water by damaged muscle also leads to intravascular dehydration, renal vasoconstriction, and decreased glomerular perfusion pressure, contributing to acute tubular necrosis [19]. Because even a single episode of acute kidney injury may increase the future CKD risk, subjects may be at risk of subsequent CKD and the associated morbidity and mortality [20]. Notably, the amputation population in the present study had a higher risk of incident ESKD than matched controls in long-term projections based on 4.3 years of follow-up, which is a novel finding.

Although the exact mechanism by which amputation contributes to ESKD development remains unclear, a possible explanation is hemodynamic changes after amputation. With a greater amputation level, the peripheral impedance increases, leading to a subsequent rise in blood pressure [21]. First, elevated blood pressure causes both decreased kidney function, a characteristic of progressive CKD, and glomerular hyperfiltration, which may be a precursor for kidney function decline [22]. Second, the redistribution of the blood flow due to amputation can lead to changes in the blood flow to visceral organs. Notably, the renal artery blood flow was reportedly increased by approximately 32% following the bilateral proximal amputation of the lower limb [21]. This could potentially result in hyperfiltration glomerulosclerosis, leading to CKD, and altered renal hemodynamics can potentially contribute to progressive CKD development. Third, commonly used medications for amputation patients include renally eliminated medications, such as adjuvant analgesics and nonsteroidal anti-inflammatory drugs during the surgical intervention and postoperatively for pain control, or anticoagulants to prevent venous thromboembolism [23,24]. Furthermore, amputation patients undergo more frequent contrast CT scans or angiography than the general population, which may lead to contrast-induced nephropathy [25]. Frequent exposure to these nephrotoxic medications and contrast media following amputation could further exacerbate renal dysfunction.

In the present study, the risk of ESKD was found to increase with the severity of disability after amputation. When the disability was severe, the risk of ESKD increased approximately 8-fold compared with the matched controls. In recent studies, higher physical activity levels were shown to be associated with better physical functioning, a lower risk of CKD, and a slower decline in the eGFR, whereas a higher sedentary time was associated with reduced kidney function and an increased CKD risk [26]. Individuals with disability tend to have limited accessibility and fewer opportunities to engage in physical activity [27]. In addition, the prevalence of traditional risk factors for ESKD, such as obesity [28], hypertension [29], and diabetes [27], are generally higher in subjects with disability than without disability. These factors may affect the metabolic environment of renal dysfunction.

According to the level of amputation, individuals with above-elbow or above-knee amputations likely represent those with the most severe disabilities, but the small sample size for these groups limits the robustness of the analysis. Except for this case, the risk of ESKD was higher for proximal amputations of both the upper and lower extremities, respectively. Compared with lower limb amputation, an upper limb deficiency is often relatively more functionally disabling due to the fine motor tasks performed by the hand and arm [30]. In fact, the risk of ESKD increased with the severity of disability in individuals with below-wrist amputation. Traumatic digit amputations are detrimental to the activities of daily life, resulting in reduced compliance with medication for comorbidities including hypertension, and affect the hand grip strength. When multiple fingers are amputated, even if the fingers have some remaining length after revision amputation, they may not be as agile in opposing and grasping as full-length fingers [31]. This reduction can contribute to chronic inflammation, a known risk factor for CKD. Furthermore, proximal upper limb amputation can exacerbate these challenges. Individuals with proximal upper limb amputation had a higher risk of ESKD than patients with other amputations. Individuals with upper limb amputation are significantly more likely to suffer depression or anxiety compared with subjects with lower limb amputation. Depression may contribute to the development of renal dysfunction due to higher rates of adverse health risk behaviors such as smoking, sedentarism, and obesity [32].

On the other hand, individuals with proximal lower limb amputation have an increased risk of ESKD comparable with subjects with distal lower limb amputation, although the above-mentioned effects appear to be more prominent for proximal amputation. Lower limb amputation causes difficulties in social activities due to mobility limitations, which can be more severe with proximal amputation than distal amputation [33]. There is a possibility that the risk of ESRD may have been underestimated due to the limited access to medical care for individuals with lower proximal amputations.

The clinical implication of the present study is that amputation has a longitudinal effect on many areas outside of the residual limb and individuals with amputation are at a significantly greater risk of incident ESKD. Even when an individual has an amputation due to trauma, their renal function can gradually worsen over time. These conditions highlight the importance of comprehensive modifiable risk factor prevention strategies including proper physical activity through rehabilitation, smoking cessation, and nutrition. Moreover, we showed that hypertension and dyslipidemia were independent risk factors for ESKD development in individuals with amputation. The management of hypertension and dyslipidemia is also crucial in preventing ESKD in this population [34]. Monitoring for renal function should be routine after amputation, and timely medical interventions should be conducted when problems are encountered.

The major strengths of the present study include the use of national claims data, which enabled a larger sample size, as well as the inclusion of age- and sex-matched controls with low attrition rates. Furthermore, adjusting for various possible confounders was possible using health screening data.

The present study had some limitations. First, information on the exact cause of amputation could not be obtained. However, all potential vascular etiologies of amputation, such as diabetic foot and Buerger’s disease, were excluded based on ICD-10 codes to focus exclusively on traumatic amputations. Second, due to the nature of the insurance claim data, detailed information regarding the etiologies of ESKD could not be obtained. Additionally, the occurrence of acute kidney injury during the perioperative period of amputation was not able to be assessed as it was not included in the current dataset. Third, the use of medications after amputation, such as antihypertensive agents, hypoglycemic agents, or nonsteroidal anti-inflammatory drugs, and the adherence to treatment [35] were not able to be considered in this study. Other potential confounders such as post-amputation behavioral changes (e.g., reduced physical activity) and an increased prevalence of comorbidities (e.g., type 2 diabetes [36], heart disease [5], and depression [14]) may have been present. Thus, even though many covariates were considered in the analysis, residual confounders remain a possibility. Fourth, when interpreting the relationship between amputation and ESKD development, caution is needed as causal relationships cannot be definitively established due to the observational study design. Lastly, the data represent outcomes in the Korean population, a homogenous ethnic population; thus, the results should not be generalized to other ethnic groups.

## 5. Conclusions

Individuals with amputation were at a significantly greater risk for ESKD than matched controls, particularly those with severe disabilities and proximal amputation. Our findings suggest that amputations should be added to the list of risk factors for developing CKD. Interventions and targeted prevention strategies addressing modifiable risk factors for ESKD should be helpful to such patients.

## Figures and Tables

**Table 1:** Baseline characteristics of the study population.

Variables	Matched Controls (n = 82,298)	Individuals with Amputation (n = 24,925)	*p* Value
Age (years)	53.4 ± 11.9	54.2 ± 12.1	<0.001
Sex (male)	64,376 (78.2)	19,007 (76.3)	<0.001
Income (lowest 20%)	14,320 (17.4)	4679 (18.8)	<0.001
Place of residence (urban)	37,568 (45.7)	8395 (33.7)	<0.001
Alcohol consumption			
None	35,974 (43.7)	11,326 (45.4)	<0.001
Mild to moderate	37,746 (45.9)	10,341 (41.5)	
Heavy	8578 (10.4)	3258 (13.1)	
Smoking status			
Never	36,491 (44.3)	11,213 (45.0)	<0.001
Former	20,830 (25.3)	5365 (21.5)	
Current	24,977 (30.4)	8347 (33.5)	
Regular physical activity	18,214 (22.1)	4387 (17.6)	<0.001
Body mass index (kg/m^2^)	24.2 ± 3.1	24.1 ± 3.2	<0.001
Systolic BP (mmHg)	124.2 ± 14.3	124.3 ± 14.7	0.504
Diastolic BP (mmHg)	77.5 ± 9.8	77.4 ± 9.9	0.145
Fasting glucose (mg/dL)	101.3 ± 24.7	101.9 ± 27.4	0.002
Total cholesterol (mg/dL)	196.7 ± 37.4	195.4 ± 37.6	<0.001
eGFR (mL/min/1.73 m^2^)	90.8 ± 51.8	92.7 ± 50.5	<0.001
Comorbidities			
Hypertension	27,722 (33.7)	8616 (34.6)	0.010
Type 2 diabetes	10,139 (12.3)	3231 (13.0)	0.007
Dyslipidemia	22,086 (26.8)	6496 (26.1)	0.015

Data are presented as number (%) or mean ± standard deviation. BP, blood pressure; eGFR, estimated glomerular filtration rate.

**Table 2:** Hazard ratios and 95% confidence intervals for the incidence of ESKD among individuals with amputation compared to the matched controls.

	Subjects (N)	Events (n)	Follow-Up Duration (Person-Years)	IR	Model 1 HR (95% CI)	Model 2 HR (95% CI)
Matched controls	82,298	100	361,584.2	0.3	1 (Ref.)	1 (Ref.)
Patients with amputation	24,925	40	106,790.9	0.4	1.36 (0.94–1.96)	1.75 (1.20–2.54)
By disability						
No disability	22,868	32	97,571.0	0.3	1.19 (0.80–1.77)	1.63 (1.09–2.45)
Disability	2057	8	9219.9	0.9	3.12 (1.52–6.41)	2.42 (1.16–5.04)
By severity of disability						
Mild disability	1672	4	7564.1	0.5	1.90 (0.70–5.16)	1.41 (0.51–3.87)
Severe disability	385	4	1655.8	2.4	8.74 (3.22–23.74)	8.22 (2.99–22.61)
By levels of amputation						
Absence of limb	41	0	93.8	–	–	–
Upper limbs						
Above the elbow	66	0	271.4	–	–	–
Between the elbow and wrist	113	2	519.7	3.8	13.73 (3.39–55.64)	17.90 (4.37–73.40)
Below the wrist *	23,962	33	102,731.8	0.3	1.17 (0.79–1.73)	1.52 (1.01–2.27)
No disability	22,200	27	94,822.9	0.3	1.04 (0.68–1.58)	1.43 (0.93–2.21)
Mild disability	1544	4	6977.7	0.6	2.06 (0.76–5.60)	1.55 (0.56–4.28)
Severe disability	218	2	931.2	2.1	7.71 (1.90–31.27)	6.91 (1.69–28.25)
Lower limbs						
Above the knee	44	0	180.1	–	–	–
Between the knee and ankle	152	2	671.0	3.0	10.83 (2.67–43.92)	5.68 (1.38–23.29)
Below the ankle	547	3	2323.3	1.3	4.64 (1.47–14.63)	5.69 (1.79–18.07)

* Due to the sufficient number of amputations below the wrist, we have included the results of the subgroup analysis according to disability. ESKD, end-stage kidney disease; IR, incidence rate (numbers of cases per 1000 person-years); HR, hazard ratio; CI, confidence interval; Model 1: crude model; Model 2: adjusted for age, sex, income level, place of residence, alcohol consumption, smoking, physical activity, BMI, eGFR, and comorbidities (hypertension, type 2 diabetes, and dyslipidemia).

**Table 3:** Baseline characteristics and factors of developing ESKD in individuals with amputation.

Variables	Amputees Who Developed No ESKD	Amputees Who Developed ESKD	*p* Value	Multivariate HR (95% CI)
Age (years)	54.1 ± 12.1	61.3 ± 10.3	<0.001	1.00 (0.97–1.04)
Sex (male)	18,975 (76.3)	32 (80.0)	0.578	2.16 (0.77–6.07)
Income (lowest 20%)	4674 (18.8)	5 (12.5)	0.309	0.88 (0.34–2.29)
Place of residence (urban)	8377 (33.7)	18 (45.0)	0.130	1.90 (0.97–3.72)
Alcohol consumption				
None	11,305 (45.4)	21 (52.5)	0.136	1 (ref.)
Mild to moderate	10,323 (41.5)	18 (45.0)		1.05 (0.50–2.23)
Heavy	3257 (13.1)	1 (2.5)		0.23 (0.03–1.79)
Smoking status				
Never	11,195 (45.0)	18 (45.0)	0.579	1 (ref.)
Former	5354 (21.5)	11 (27.5)		1.31 (0.53–3.21)
Current	8336 (33.5)	11 (27.5)		1.32 (0.53–3.28)
Regular physical activity	4383 (17.6)	4 (10.0)	0.207	0.47 (0.16–1.34)
Body mass index (kg/m^2^)	24.1 ± 3.2	22.8 ± 2.9	0.010	0.80 (0.66–0.98)
Systolic BP (mmHg)	124.3 ± 14.7	132.2 ±16.5	<0.001	1.02 (0.99–1.05)
Diastolic BP (mmHg)	77.4 ± 9.9	80.4 ± 9.4	0.052	0.99 (0.95–1.04)
Fasting glucose (mg/dL)	101.8 ± 27.1	142.1 ± 86.0	<0.001	1.01 (1.00–1.01)
Total cholesterol (mg/dL)	195.4 ± 37.6	192.2 ± 44.8	0.594	1.00 (0.99–1.00)
eGFR (mL/min/1.73 m^2^)	92.8 ± 50.5	53.1 ± 22.9	<0.001	0.94 (0.93–0.95)
Comorbidities				
Hypertension	8582 (34.5)	34 (85.0)	<0.001	5.55 (2.00–15.39)
Type 2 diabetes	3210 (12.9)	21 (52.5)	<0.001	1.70 (0.75–3.89)
Dyslipidemia	6473 (26.0)	23 (57.5)	<0.001	2.49 (1.19–5.21)

Data are presented as number (%) or mean ± standard deviation. ESKD, end-stage kidney disease; HR, hazard ratio; CI, confidence interval; BP, blood pressure; eGFR, estimated glomerular filtration rate.

## Data Availability

The datasets presented in this article are not readily available because restrictions apply to the availability of these data, which were used under license for this study. Requests to access the datasets should be directed to https://nhiss.nhis.or.kr/en/z/a/001/lpza001m01en.do with the permission of the Korean National Health Insurance Service.

## Supplementary Materials

The following supporting information can be downloaded at: https://www.mdpi.com/article/10.3390/healthcare13010080/s1, Table S1. Definitions of severity degree in upper extremity amputation; Table S2. Definitions of severity degree in lower extremity amputation.
