# Supplementary material for: Increased Risk of End-Stage Kidney Disease After Traumatic Amputation: Nationwide Cohort Study
Source: Healthcare (Basel). 2025 Jan 4;13(1):80. doi: 10.3390/healthcare13010080 (PMC11720595; doi:10.3390/healthcare13010080)
Supplement: Supplementary file 1 [file healthcare-13-00080-s001.zip › healthcare-3347165-supplementary.pdf]

Table S1. Definitions of severity degree in upper extremity amputation

Grade	Definitions
1	Amputation above the wrist joint of both arms
2	Amputation of both thumbs above the IP joint and all 2nd to 5th fingers above the PIP joint
	Amputation above the elbow joint of one arm
3	Amputation of both thumbs above the IP joint and the 2nd finger above the PIP joint
	Amputation of one thumb above the IP joint and all 2nd to 5th fingers above the PIP joint
4	Amputation of both thumbs above the IP joint
	Amputation of one thumb above the IP joint and the 2nd finger above the PIP joint
	Amputation of one thumb above the IP joint and another two fingers above the PIP joint
5	Amputation of one thumb above the IP joint and another finger above the PIP joint
	Amputation of one thumb above the MCP joint
	Amputation of three fingers including the 2nd finger above the PIP joint
6	Amputation of one thumb above the IP joint
	Amputation of two fingers including the 2nd finger above the PIP joint
	Amputation of all 3rd to 5th fingers of one hand above the PIP joint

IP, Interphalangeal; PIP, proximal interphalangeal; MCP, metacarpophalangeal

Adapted from Kim, M.; Jung, W.; Kim, S.Y.; Park, J.H.; Shin, D.W. The Korea National Disability Registration System. *Epidemiol Health* **2023**, *45*, e2023053, doi:10.4178/epih.e2023053.

Table S2. Definitions of severity degree in lower extremity amputation

Grade	Definitions
**1**	Amputation of both legs above the knee joint
**2**	Amputation of both legs above the ankle joint
**3**	Amputation of both legs above the transverse tarsal joint (Chopart joint)
	Amputation of one leg above the knee joint
**4**	Amputation of both legs above the tarsometatarsal joint (Lisfranc joint)
	Amputation of one leg above the ankle joint
**5**	Amputation of both big toes above the interphalangeal joint and the 2nd to 5th toes above the proximal interphalangeal joint
	Amputation of one leg above the transverse tarsal joint (Chopart joint)
**6**	Amputation of one leg above the tarsometatarsal joint (Lisfranc joint)

Adapted from Kim, M.; Jung, W.; Kim, S.Y.; Park, J.H.; Shin, D.W. The Korea National Disability Registration System. *Epidemiol Health* **2023**, *45*, e2023053, doi:10.4178/epih.e2023053.
